# Calcineurin/NFAT Signaling Represses Genes *Vamp1* and *Vamp2* via PMCA-Dependent Mechanism during Dopamine Secretion by Pheochromocytoma Cells

**DOI:** 10.1371/journal.pone.0092176

**Published:** 2014-03-25

**Authors:** Michalina Kosiorek, Ludmila Zylinska, Krzysztof Zablocki, Slawomir Pikula

**Affiliations:** 1 Department of Biochemistry, Nencki Institute of Experimental Biology, PAS, Warsaw, Poland; 2 Laboratory of Neurogenetics, Department of Neurodegenerative Disorders, Mossakowski Medical Research Centre PAS, Warsaw, Poland; 3 Department of Molecular Neurochemistry, Medical University, Lodz, Poland; Univ. Kentucky, United States of America

## Abstract

**Background:**

Plasma membrane Ca^2+^-ATPases (PMCA) extrude Ca^2+^ ions out of the cell and contribute to generation of calcium oscillations. Calcium signaling is crucial for transcriptional regulation of dopamine secretion by neuroendocrine PC12 cells. Low resting [Ca^2+^]_c_ in PC12 cells is maintained mainly by two Ca^2+^-ATPases, PMCA2 and PMCA3. Recently, we found that Ca^2+^ dependent phosphatase calcineurin was excessively activated under conditions of experimental downregulation of PMCA2 or PMCA3. Thus, the aim of this study was to explain if, via modulation of the Ca^2+^/calcineurin-dependent nuclear factor of activated T cells (NFAT) pathway, PMCA2 and PMCA3 affect intracellular signaling in pheochromocytoma/neuronal cells/PC12 cells. Secondly, we tested whether this might influence dopamine secretion by PC12 cells.

**Results:**

PMCA2- and PMCA3-deficient cells displayed profound decrease in dopamine secretion accompanied by a permanent increase in [Ca^2+^]_c_. Reduction in secretion might result from changes in NFAT signaling, following altered PMCA pattern. Consequently, activation of NFAT1 and NFAT3 transcription factors was observed in PMCA2- or PMCA3-deficient cells. Furthermore, chromatin immunoprecipitation assay indicated that NFATs could be involved in repression of *Vamp* genes encoding vesicle associated membrane proteins (VAMP).

**Conclusions:**

PMCA2 and PMCA3 are crucial for dopamine secretion in PC12 cells. Reduction in PMCA2 or PMCA3 led to calcium-dependent activation of calcineurin/NFAT signaling and, in consequence, to repression of the *Vamp* gene and deterioration of the SNARE complex formation in PC12 cells.

## Introduction

Pheochromocytoma is a tumor characterized by an excessive catecholamine secretion [1]. One of the catecholamines excessively secreted during progression of this tumor, is dopamine. This is a neurotransmitter and neurohormone known to be involved in a variety of processes in the brain, including cognition, learning, attention, reward system, control of emotions and motor coordination [2]. An impaired dopaminergic signaling has been observed in several neurological disorders; i.e. Parkinson's disease, Alzheimer's disease, schizophrenia, or depression [2]–[4]. Dopamine is released from neurons and neuroendocrine cells by Ca^2+^-dependent exocytosis, that engages complex molecular regulatory mechanisms. Therefore, in this study, using PC12 cells as a model, we focused on a^2+^-dependent signaling during dopamine secretion in dopaminergic tumor pheochromocytoma.

Maintenance of calcium homeostasis is critical for signaling during dopamine secretion. Cytosolic concentration of calcium ions ([Ca^2+^]_c_) is controlled in PC12 cells by a complex network of calcium transporters. The isoforms of plasma membrane Ca^2+^-ATPases (PMCAs) are important elements of this network [5]. PMCAs pump Ca^2+^ ions out of the cell to maintain low [Ca^2+^]_c_. PC12 cells express four isoforms of PMCA, encoded by independent genes: *Atp2b1*, *Atp2b2*, *Atp2b3*, and *Atp2b4* [6]. PMCA1 and PMCA4 are ubiquitous isoforms, expressed in most mammalian tissues [6]–[8]. PMCA2 is specifically expressed in excitable cells, including neurons and neuroendocrine cells, but is also playing important roles in intracellular transport in epithelial cells of the mammary gland [6]–[9]. PMCA3, similarly to PMCA2, is expressed in neuronal and neuroendocrine cells, including PC12 cells, but also is found in such specialized organs like placenta [6]–[8]. Both PMCA2 and PMCA3 are characterized by higher affinity for calcium ions and better efficiency of calcium removal [6]–[8], [10]. Tissue specificity and functional diversity of PMCA isoforms lead to a question about their role in dopamine secretion.

Signaling during dopamine secretion might be affected at various steps, i.e. during vesicular traffic, vesicle docking or membrane fusion [11], [12]. Membrane fusion requires assembly of the SNARE complex (soluble NSF-attachment protein receptors) composed of v-SNARE (vesicular) and t-SNARE (target) proteins. In neural cell types, the v-SNARE are vesicle-associated membrane proteins (VAMP) and the t-SNARE are syntaxins and SNAP-25 (synaptosomal-associated protein 25) [13]. The molecular machinery of exocytosis is well known, but the mechanisms determining the expression pattern of the secretory components have not been fully understood yet [14]. In view of this information, searching for new transcription factors involved in the regulation of excessive dopamine secretion from dopaminergic tumors is justified and may extend our knowledge of the mechanisms of neurotransmitter secretion. It may also be important in understanding the pathophysiology of some neuronal diseases.

Previously, we demonstrated that the activity of Ca^2+^-dependent phosphatase, calcineurin, increased in PC12 cells as a consequence of downregulation of PMCA2 and PMCA3 isoforms [15]. One of the main substrates of calcineurin is the transcriptional nuclear factor of activated T-cells (NFAT). NFATs constitute a family of transcription factors related to the Rel/NF-κB family that is composed of five isoforms. Upon a rise in [Ca^2+^]_c_ NFAT isoforms 1–4 become activated by calcineurin-mediated dephosphorylation of several serine residues that uncovers the nuclear localization signal sequence and is prerequisite for its nuclear localization [16]. NFAT5 is a calcineurin -independent and cAMP-dependent NFAT isoform [17]. The role of NFATs in the neurological system is not fully understood. A ubiquitous isoform, NFAT1, has been discovered as a transcriptional factor involved in regulation of secretion of interleukins during activation of T cells [18]. In neural cells, NFAT3 was found to play an important role in axon outgrowth, neuronal maturation and survival via engagement in signaling for neuronal apoptosis [19]–[21].

Summarizing, the aim of this study was to investigate the role of neurospecific PMCA2 and PMCA3 isoforms, specialized in rapid and efficient calcium removal, in dopamine secretion by neuroendocrine PC12 cells. More specifically, in this report we tested the hypothesis that PMCAs might mediate calcineurin/NFAT signaling during the secretory response.

## Materials and Methods

### Cell culture and cell lines

PC12 cell line was obtained by courtesy of prof. Ludmila Żylińska from the Department of Molecular Neurochemistry, Medical University of Lodz (purchased from American Type Culture Collection, ATCC No.: CRL-1722). PC12 cells were cultured in RPMI-1640 medium (Sigma Aldrich, USA) with 10% heat-inactivated horse serum and 5% heat-inactivated fetal bovine serum (Gibco, Invitrogen), in a humidified atmosphere of 5% CO_2_/95% air at 37°C. In some experiments the inhibitor of all isoforms of NFAT (1 µM 11R-VIVIT) (Calbiochem Merck Chemicals, Germany) was added for 48 h to the cell culture. Cell lines deficient in PMCA2 (_2) or PMCA3 (_3) were established as described previously [15], [22]. Control cells (C) were transfected with empty pcDNA3.1(+) plasmid. Stable cell lines were obtained by a 4-week selection with 1 mg/ml G418 and used for the experiments at passages 15–30. Cells were tested in terms of cell cycle and apoptosis index, and none of used conditions (5 mM KCl, 59 mM KCl or 1 µM 11R-VIVIT) did affected the measured parameters (Figure S5).

### RNA isolation, reverse transcription, quantitative PCR (qPCR) and RT-PCR

Total RNA was isolated using TRIzol Reagent (Invitrogen). RNA aliquots of 5 µg were subjected to reverse transcription reaction with reverse transcriptase MuMLV (Promega), according to the manufacturer's protocol. The obtained cDNA was subjected to real-time quantitative PCR (qPCR) with the SYBR Green reagent (Applied Biosystem) according to the manufacturer's protocol. qPCR data were normalized to *Gapdh* expression and calculated according to the ΔΔC_T_ method [23]. The calculations for 11R-VIVIT treated cells were carried out according to a modified ΔΔC_T_ method as follows: ΔΔC_T_ = ΔC_11R-VIVIT-treated_ - ΔC_non-treated_. PMCA isoforms expression was also verified by RT-PCR, as described previously [23]. All primers were designed for the *R. norvegicus* genome using the GenScript Primer Design Tool (USA) (Table 1).

**Table 1:** Primers designed for *Rattus norvegicus* genome using GenScript real-time qPCR design tool.

gene name	sequence (5′→3′)	Strand	Tm °C	amplicon size (bp)	NCBI number
*Atp2b2* a **^,^** c	TTGCTGTCAGGAACTCATGT	F	56.76	79	NM_012508.5
	TGCCAGTTTGAGAGTTGACA	R	57.95		
*Atp2b3* a **^,^** c	GAAAGCAGGATTGGTGATGT	F	57.58	164	NM_133288.1
	CAACCAACACAGTGACTCCA	R	58.06		
*Snap25* a **^,^** c	TTGTTGATCACCATTTCCCT	F	57.84	200	NM_030991.2
	CAGAGGAGACAGGAGGGATT	R	58.26		
*Stx1a* a **^,^** c	TACAACGCCACTCAGTCAGA	F	57.98	155	NM_053788.2
	GAGTCCATGATGATCCCAGA	R	58.37		
*Vamp1* a **^,^** c	GGGTTTCCATTGTGTCTGTC	F	57.81	105	NM_013090.2
	ATCTGTCACATGCCTTTGGT	R	58.00		
*Vamp2* a **^,^** c	TGCACCTCCTCCAAATCTTA	F	58.30	138	NM_012663.2
	CGATCATCCAGTTCCGATAG	R	58.12		
*Gapdh* a **^,^** c	GAACATCATCCCTGCATCCA	F	51.79	78	NG_028301.1
	CCAGTGAGCTTCCCGTTCA	R	53.85		
*Gapdh* b	GATGACATCAAGAAGGTGGTGAAGCA	F	58.00	500	NG_028301.1
	TCCACCACCCTGTTGCTGTAGCC	R	58.50		
*Atp2b2* b	GCTCGAGTTCTGCTTGAGCGC	F	65.00	602	NM_012508.5
	AAGATCCACGGCGAGCGTAAC	R	63.00		
*Atp2b3* b	ATGCACCACCTGGAGAGGAAAG	F	64.00	302	NM_133288.1
	CAGGCAGAAGATCTCCGTATTTG	R	63.00		

a primers used for qPCR.

b primers used for RT-PCR.

c primers used for chromatin immunoprecipitation assay.

### [Ca^2+^]_c_ measurements

[Ca^2+^]_c_ in resting (5 mM KCl) or in stimulating Locke's solution (59 mM KCl) was measured with a RF-5301PC Spectrofluorometer (Shimadzu, Japan) using Fura-2 AM (Molecular Probes) as already described [15]. [Ca^2+^]_c_ was calculated according to the method described in Grynkiewicz et al [24].

### RP-HPLC measurements of dopamine secretion

The amount of dopamine secreted by PC12 cells was measured by reverse phase-high performance liquid chromatography (RP-HPLC) as described previously [15]. For the experiments, PC12 cells grown at 50–60% confluence were incubated in resting (5 mM KCl) or in stimulating Locke's solution (59 mM KCl) for 1, 5, 10, 15, 20 or 30 min.

### Subcellular fractionation

PC12 cells (10^7^) were incubated for 10 min in resting (5 mM KCl) or stimulating (59 mM KCl) Locke's solution. Then, cells were subjected to subcellular fractionation as described previously [25]. Protein concentration was determined in the obtained fractions with the Bradford reagent (Bio-Rad). Sucrose gradient linearity was determined in the obtained fractions using a refractometer. Dopamine content was analyzed by subjecting the fractions to RP-HPLC analysis. The subcellular compartments were identified by subjecting the fractions to immunoblotting for the presence of the following protein markers: SNAP-25 and α1-Na^+^/K^+^-ATPase for plasma membrane, 58K and GM130 for *trans*- and *cis*-Golgi network, and Rab3A and dopamine β-hydroxylase (DBH) for immature secretory vesicles. Synaptophysin, a marker of small synaptic vesicles, was additionally used to ascertain the prevalence of large dense core vesicles (LDCV) in the obtained fractions (immunoblotting of the fractions is shown in Fig. S2).

### Total cell lysate preparation

Cells were harvested and washed with PBS. Cells were incubated for 40 min in an ice-cold lysis buffer containing 10 mM Tris-HCl (pH 7.5) 1% Triton X-100, 1 mM dithiothreitol, 1 mM PMSF, 10 mM NaF, 2 mM Na_3_VO_4_ and protein inhibitor cocktail (PIC, Sigma Aldrich), centrifuged at 800× g for 5 min at 4°C to obtain total cell lysates which were stored at −20°C until analyzed. Protein concentration was determined with the Bradford reagent (Biorad).

### Isolation of nuclear fractions

Nuclear fractions were obtained by a method based on different concentrations of Triton X-100 and sucrose, as described by Blobel and Potter [26]. Cells (4×10^6^) were incubated in Locke's solution with 5 or 59 mM KCl for 10 min. Cytosolic (glyceraldehyde 3-phosphate dehydrogenase, GAPDH) and nuclear (poly ADP ribose polymerase, PARP) markers were used to verify purity of the fractions.

### Luciferase Reporter Assay

Luciferase reporter plasmid with NFAT-dependent promoter (pGL3-NFAT-luc), *Renilla* luciferase control plasmid (pRL-SV40), promoter less plasmid, pGL3-luc, and plasmid overexpressing NFAT (pNFAT+/+) were gifts from Dr. Wieslawa Lesniak from the Nencki Institute of Experimental Biology. PC12 cells (2×10^5^) were transfected with X-tremeGENE Transfections Reagent (Roche Applied Science, Germany) with the following plasmid combination: pGL3-NFAT-luc with pRL-SV40, pGL3-luc with pRL-SV40 (negative control), pNFAT+/+, with pGL3-NFAT-luc and with pRL-SV40 (positive control). Cells were harvested 48 h after transfection and lysed in lysis reagent (Thermo Scientific Pierce). Firefly and *Renilla* luciferase activities were assayed with Pierce *Renilla*-Firefly Luciferase Dual Assay Kit (Thermo Scientific Pierce). The luminescent signal from *Renilla* luciferase was measured at λ_max_ = 535 nm and from firefly luciferase at λ_max_ = 613 nm. The working solution contained substrates for both luciferases (coelenterazine and D-luciferin), and the reactions occurred simultaneously with flash-type kinetics. The luminescent signals were spectrally resolvable using filters. The activity of NFAT was determined based on the luminescence signal from firefly luciferase and standardized to the signal from *Renilla* luciferase. The luminescence emission was determined by a SpectraMax M5e Microplate Reader (Molecular Devices, Sunnyvale, California, United States). The efficiency of transfection was verified by transfections with plasmid overexpressing EGFP and determined as 20% on the basis of cell counting under a fluorescent microscope.

### Immunoblotting

Total cell lysates, nuclear or subcellular fractions were subjected to immunoblotting as described previously [13]. The antibodies used for the experiments are described in (Table 2). Band intensities were analyzed densitometrically by the Ingenius Bioimaging with the Gene Tools 3.06 software (Syngene, UK).

**Table 2:** Primary antibodies raised against *Rattus norvergicus*.

protein name	gel band size (kDa)	host	supplier	dilution
PMCA2	127 (2b) 133 (2a)	rabbit	Affinity Bioreagents	1∶2000
PMCA3	127 (3a, 3b)	rabbit	Affinity Bioreagents	1∶1000
NFAT1	120 (140 phospho)	mouse	Abcam	1∶1000
NFAT1	120	rabbit	Cell Signaling (for ChIP)	1∶50
NFAT3	120	rabbit	Cell Signaling (for ChIP)	1∶50
PARP	116	mouse	Enzo Technologies	1∶1000
GAPDH	36	mouse	Millipore Chemicon	1∶1000
β-actin	43	mouse	Calbiochem	1∶5000

### Confocal microscopy of SNAP-25 and VAMP2 and FRET measurements

The immunocytochemical stainings were performed with primary antibodies: mouse monoclonal to SNAP-25 (Santa Cruz) and rabbit polyclonal to VAMP2 (Abcam). Secondary antibodies were Alexa Fluor 488 goat anti-rabbit and Alexa Fluor 546 goat anti-mouse (Invitrogen). The obtained images were used for the study of SNAP-25 and VAMP2 interaction by Förster resonance energy transfer (FRET) with a TCS SP5 Leica Confocal Microscope, as described previously [15].

### Chromatin immunoprecipitation (ChIP)

PC12 cells (2×10^7^) were cross-linked with 1% formaldehyde for 10 min at room temperature. Cross-linking was stopped by adding 125 mM glycine at 4°C. Cells were solubilized in a buffer containing 10 mM Tris-HCl, 1% Triton X-100, 1% sodium deoxycholate, 1 mM PMSF and PIC (pH 8.0) for 10 min at 4°C. Pellets obtained by centrifugation at 1000× g for 5 min were suspended in RIPA buffer and sonicated using a Bioruptor Sonicator (Diagenode, Belgium) to shear chromatin into 500 bp fragments. Sonicated chromatin was subjected to immunoprecipitation using agarose beads ChIP-Grade (Cell Signaling), blocked with 1% bovine albumin and 1% salmon sperm DNA, and antibodies recognizing NFAT1 (Cell Signaling) or NFAT3 (Cell Signaling). DNA-protein complexes were eluted with 100 mM sodium acetate and 1% SDS for 30 min and incubated with RNase for 6 h at 65°C and proteinase K o/n at 45°C. DNA was isolated using the phenol/chloroform/isoamyl reagent (Sigma Aldrich) and subjected to qPCR analysis as described above. The qPCR data, were expressed as fold of change (2^−ΔΔC^) calculated from the difference: ΔC_T_ of output (DNA immunoprecipitated with NFAT1 or NFAT3) – ΔC_T_ of input (total DNA).

### Statistical analysis

All data are presented as mean ± SEM of n observations. Data were analyzed by Student's t test at 95% or 99% confidence. Two-way ANOVA test was used at 95% or 99% confidence for comparison between all experimental groups (3 cell lines) for the experiments with 1 µM 11R-VIVIT. For quantitative PCR statistics, the nonparametric paired Wilcoxon signed rank test was used at 95% or 99% confidence.

## Results

### Downregulation of PMCA2 and PMCA3 alters [Ca^2+^]_c_ in PC12 cells

**Figure 1:**
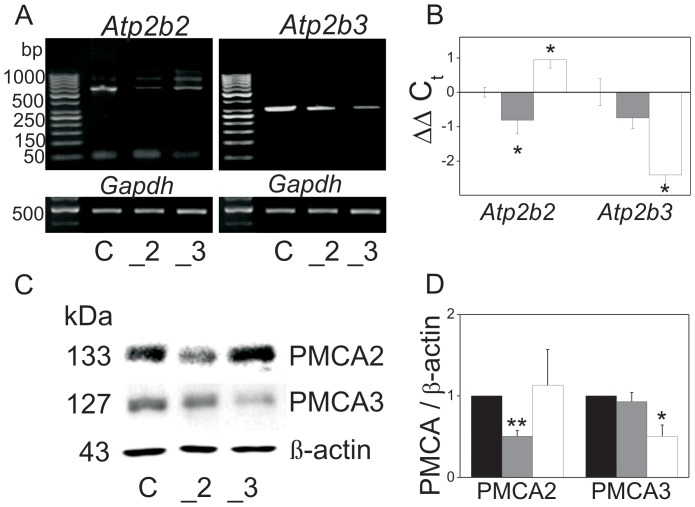
Characterization of PMCA2- or PMCA3-deficient PC12 cells. Expression of PMCA2 (*Atp21b2*) and PMCA3 (*Atp2b2*) in PC12 was determined by RT-PCR (**A**). The RT-PCR results were validated by qPCR (**B**). PMCA2 and PMCA3 protein content was analyzed by immunoblotting, standardized to β-actin level (**C**). The immunoblotted bands of PMCA2 or PMCA3 were quantified densitometrically, standardized to β-actin and normalized to control cells, expressed as y = 1 (**D**). Bars represent mean values ± SEM. Student's t-test was used for comparison of control cells with PMCA2- or PMCA3-reduced cells (n = 6). Wilcoxon test was used for ΔC_t_ from qPCR data (n = 3) for comparison of control cells (ΔC_t_ expressed as y = 0) with PMCA2- or PMCA3-reduced cells (n = 3). *P≤0.05, **P≤0.01. Bars and symbols: filled – control cells (C), gray – PMCA2-deficient cells (_2), open – PMCA3-deficient cells (_3).

**Figure 2:**
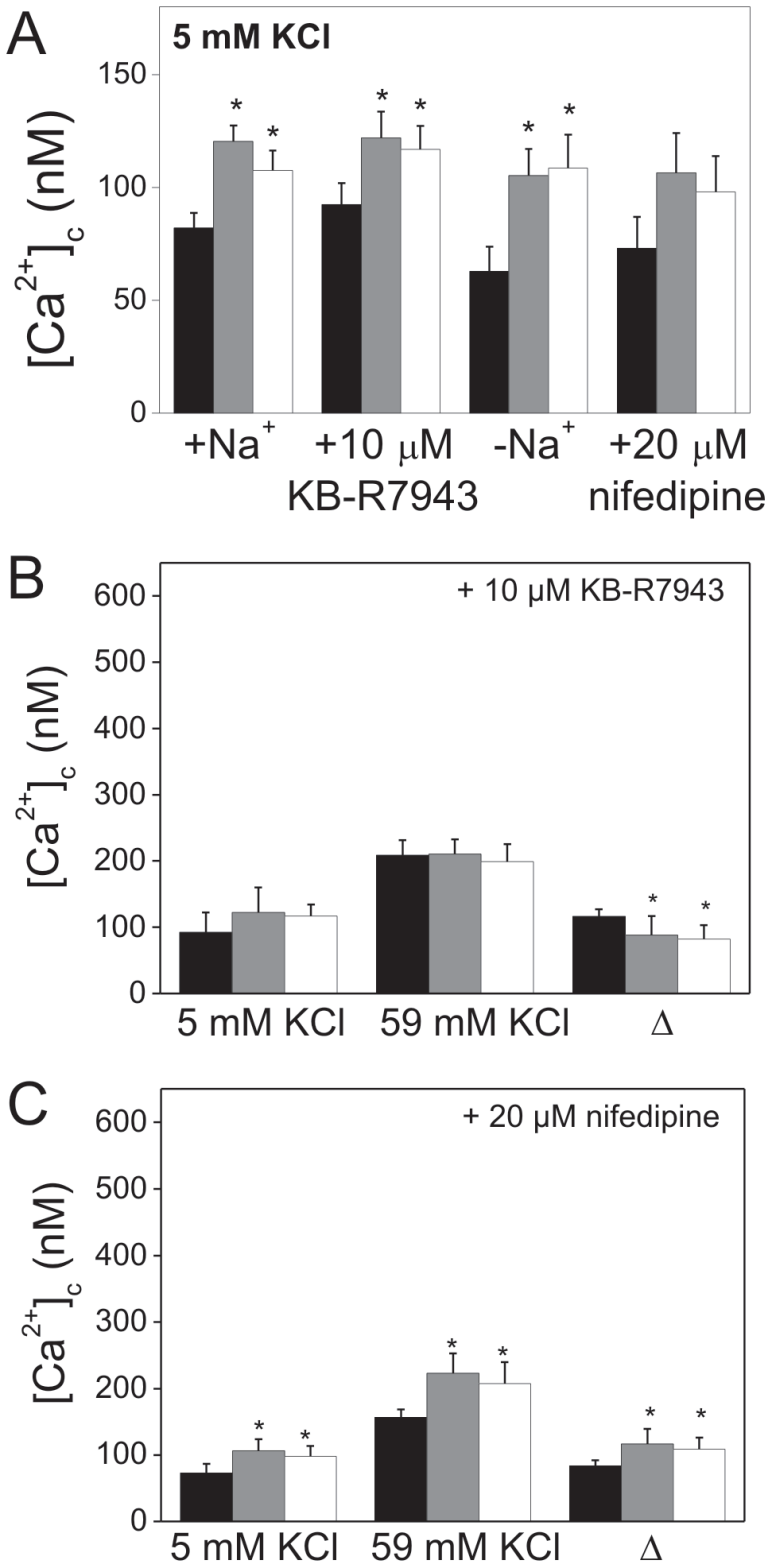
Effects of PMCA2 or PMCA3 deficiency in PC12 cells on [Ca^2+^]_c_ under resting and depolarizing conditions. Measurements of [Ca^2+^]_c_ were performed with Fura-2 AM. [Ca^2+^]_c_ was measured under resting conditions (5 mM KCl) in the presence of sodium ions or not (+Na^+^ or −Na^+^), 10 µM KB-R7943 (NCX inhibitor) or 20 µM nifedipine (VDCC inhibitor) (**A**). The effect of 10 µM KB-R7943 (**B**) or 20 µM nifedipine (**C**) on Ca^2+^]_c_ under resting and stimulatory conditions. The statistical analysis was performed for n>10 measurements and calcium entry is expressed as Δ of [Ca^2+^]_c_ values between depolarizing (59 mM KCl) and resting (5 mM KCl) conditions. Bars represent mean values ±SEM. Student's t-test was used for comparison of control cells with PMCA2- or PMCA3-reduced cells. *P≤0.05, **P≤0.01, n>10. Bars and symbols: filled – control cells (C), gray – PMCA2-deficient cells (_2), open – PMCA3-deficient cells (_3).

Experimental downregulation of PMCA2 and PMCA3 in PC12 cells [15], [22] resulted in reduction of *Atp2b2* and *Atp2b3* transcripts as tested by RT-PCR (Fig. 1A) and by qPCR (Fig. 1B) revealing a significant decrease of 1.91±0.5 and 2.97±0.6 fold (2^−ΔΔCt^), respectively. Furthermore, a reduced mRNA transcript content of PMCA2 or PMCA3 isoform corresponded to an approximate 40% drop of the respective protein content (Fig. 1C, D). The qPCR results are in agreement with the protein level, showing that in PMCA3-deficient cells there was a slight increase in a protein content of the PMCA2 isoform. The RT-PCR assays were verified by performing additional experiments with the use of primers designed to recognize different splicing sites and to detect different splicing products (according to [27]). The results of RT-PCR-alternative splicing for Atpb2b (Figure S6) showed that the increase in PMCA2 expression in PMCA3-deficient cells concerned especially the PMCA2× splicing form. As we have already reported [15], downregulation of PMCA2 or PMCA3 isoforms resulted in a permanent elevation of [Ca^2+^]_c_ under resting conditions (5 mM KCl) and an increase in calcium influx upon plasma membrane depolarization in the presence of 59 mM KCl [15]. These changes were linked with the reduced level of selected PMCA isoforms. They were not compensated either by sodium-calcium exchanger activity in the forward mode or calcium fluxes through voltage gated calcium channels (at the resting state) (Fig. 2A). As it is commonly known, precisely controlled calcium signaling is fundamental for regulation of gene expression. Thus, our findings point to the contribution of PMCAs to calcium-dependent signaling needed for gene expression and suggest that this process might be affected, especially at the resting state, due to deficiency in certain PMCA isoforms. Calcium measurements suggested that PMCA2 and PMCA3 are indispensable for the maintenance of low, stable calcium ion concentration in PC12 cells at the resting state. Interestingly, a decrease in PMCA2 or PMCA3 content resulted also in abnormal elevated calcium influx upon stimulation. This was probably due to an inefficient calcium removal by the disabled PMCAs set as well as due to stimulation of NCX (in the reverse mode) and VDCC. It must be stressed that upon depolarization in the presence of NCX inhibitor (10 µM KB-R941 maximal [Ca^2+^]_c_ values were the same in all tested cell lines. Moreover, a factual calcium influx, expressed as the differences between [Ca^2+^]_c_ upon depolarization and [Ca^2+^]_c_ under resting conditions (Δ values), was similar in all cell lines, suggesting that NCX contribution to elevated calcium influx in PMCA2- or PMCA3-deficient cells was higher than in control cells. On the other hand, the persisting difference in calcium influx (expressed as Δ values) between control and PMCA-reduced cell lines in the presence of L-type VDCC inhibitor (20 µM nifedipine, Fig. 2C) implicate that L-type VDCC contribution to elevated calcium entry upon plasma membrane depolarization was less significant than that of NCX. The expression level of NCX isoforms and different types of VDCC was verified by qPCR and immunoblotting, and shown to be upregulated in cells with reduced level of PMCA2 or PMCA3 (Fig. S1). This could explain increased contribution of NCX and VDCC to massive calcium entry upon depolarization or may reflect more refined regulatory mechanism.

### Over-activation of NFATs in PMCA2- or PMCA3-deficient PC12 cells

**Figure 3:**
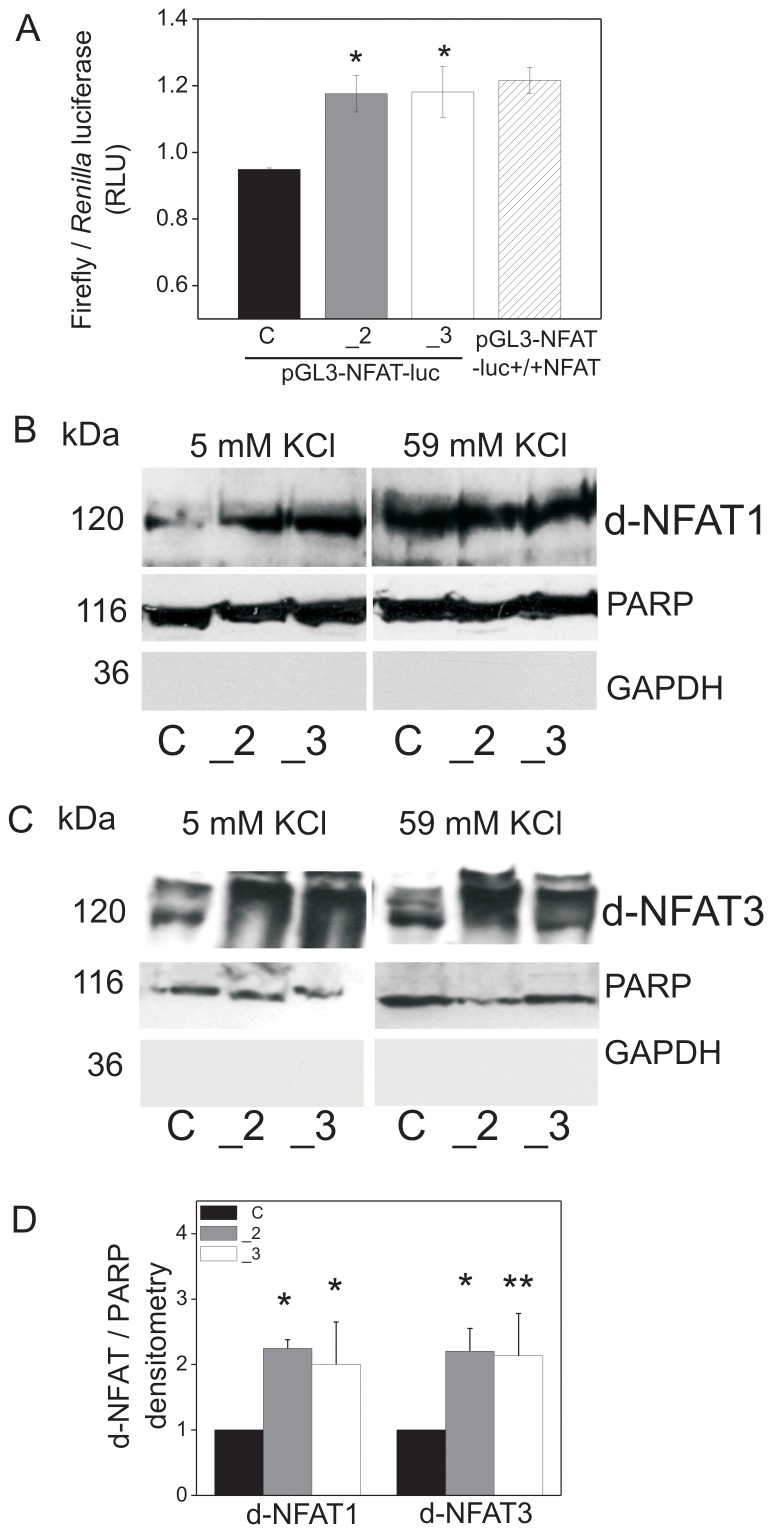
Activity and nuclear translocation of NFAT1 and NFAT3 in PMCA2- or PMCA3-deficient PC12 cells. PC12 cells were transfected with plasmids carrying firefly luciferase gene under the control of NFAT-dependent promoter (pGL3-NFAT-luc) and with reference plasmids with *Renilla* luciferase (pRL-SV40); the negative control was a promoter less plasmid pGL3-luc. Simultaneously, PC12 cells were transfected with plasmids overexpressing NFAT (pGL3-NFAT-luc-NFAT+/+) (positive control). NFAT activity was determined with luciferase reporter dual assay as described in methods. Bars represent mean values ± SEM. Student's t-test was used for comparing control cells with PMCA2- or PMCA3-reduced cells. *P≤0.05 (**A**). Nuclear fractions, obtained from PC12 cells incubated with 5 mM KCl or 59 mM KCl for 10 min, were analyzed in terms of the content of dephosphorylated NFAT1 (d-NFAT1) (**B**) and dephosphorylated NFAT3 (d-NFAT3) The purity of fractions was tested by immunoblotting for cytosolic (GAPDH) and nuclear (PARP) markers. (**C**). Mean values ± SEM from 3 experiments shown in C. All immunoblots were quantified densitometrically, standardized to the content of nuclear poly (ADP-ribose) polymerase (PARP) and normalized to control cells (y = 1). Student's t-test was used in the densitometry for comparison of control cells with PMCA2- or PMCA3-reduced cells (n>3) (**D**). Student's t-test was used for comparison control cells with PMCA2- or PMCA3-reduced cells. *P≤0.05. Symbols: control cells (C), PMCA2-deficient cells (_2),PMCA3-deficient cells (_3).

Altered calcium signaling as shown earlier [15], especially under resting conditions, might lead to deregulation of the transcriptional machinery. Since over-activation of calcineurin in PMCA2- or PMCA3 reduced cells was reported previously [15], in this study we tested subcellular and activity of the Ca^2+^/calcineurin-dependent transcription factor, NFAT. Dephosphorylation of serine-proline repeats at the N-termini of NFAT by calcineurin unmasks the nuclear localization signal (NLS) sequence and leads to NFAT translocation to the nucleus [16]. Thus, in this report NFAT activation was estimated by its dephosphorylation state in nuclei (the content of dephosphorylated protein of the ubiquitous isoform NFAT1 and neural isoform NFAT3) as well as by the luciferase reporter dual assay. The data obtained by the latter method demonstrated that NFAT activation was significantly higher in PMCA2- or PMCA3-deficient cells comparing to control cells, and similar in comparison to the positive control cells overexpressing NFAT (pGL3-NFAT-luc-+/+NFAT transfected cells) (Fig. 3A). In agreement with the results of the luciferase reporter dual assay, was the amount of dephosphorylated NFAT1 in nuclear fractions of PMCA2 or PMCA3-deficient cells under resting conditions. Upon stimulation the nuclear content of NFAT1 was similar in all cell lines (Fig. 3B). This suggested that altered calcium signaling upon PMCA2- or PMCA3-deficiency may lead to the activation of the ubiquitous NFAT1 isoform already at the resting state, but in stimulated cells the degree of NFAT1 activation seemed to be independent of PMCA isoform composition. The NFAT3 isoform nuclear level was similar to that of NFAT1 under resting conditions, higher in PMCA2- and PMCA-deficient cells, comparing to control cells. Contrary to NFAT1, under stimulation, NFAT3 nuclear content was significantly higher in PMCA2 or PMCA3-deficient cells, in comparison to controls (Fig. 3C and 3D). These data suggest that NFAT3 activation was dependent on the proportion between PMCA isoforms both at the resting state and upon stimulation via plasma membrane depolarization of PC12 cells. It should be underlined at this point that NFAT3 appeared not to be activated in response to 59 mM KCl in control cells. This might be explained and supported by several data. For instance, an addition of high concentration of KCl together with serum deprivation decreased the number of neurons expressing nuclear NFAT3 [19]. Electrically evoked elevation of [Ca^2+^]_c_ in neurons rapidly and strongly activated NFAT2, whereas activation of NFAT3 required a coincident increase in [Ca^2+^]_c_ and suppression of glycogen synthase kinase 3β (GSK3β) (NFAT-activating kinase), with differences in the serine-proline-containing region giving rise to these distinct activation properties of NFAT2 and NFAT3 [28]. Finally, Ca^2+^ entry via T-type VDCC may block pathophysiological signaling pathways leading to hypertrophy in cardiomyocytes, putatively via NFAT3 signaling [29]. Since control cells in our studies exhibited lower content of L type and T type VDCC than PMCA2- or PMCA3-deficient cells (Fig. S1), it is very likely that this affected NFAT3 activation response. The above data suggest that activation of NFAT3 may differ depending on intracellular conditions. In our other studies we also found that control cells exhibited different parameters of plasma membrane depolarization/different resting membrane potential which could result in preferential opening of distinct VDCC types than in PMCA2- or PMCA3-deficient cells.

### Dynamics of dopamine secretion in PMCA2- or PMCA3-deficient PC12 cells

**Figure 4:**
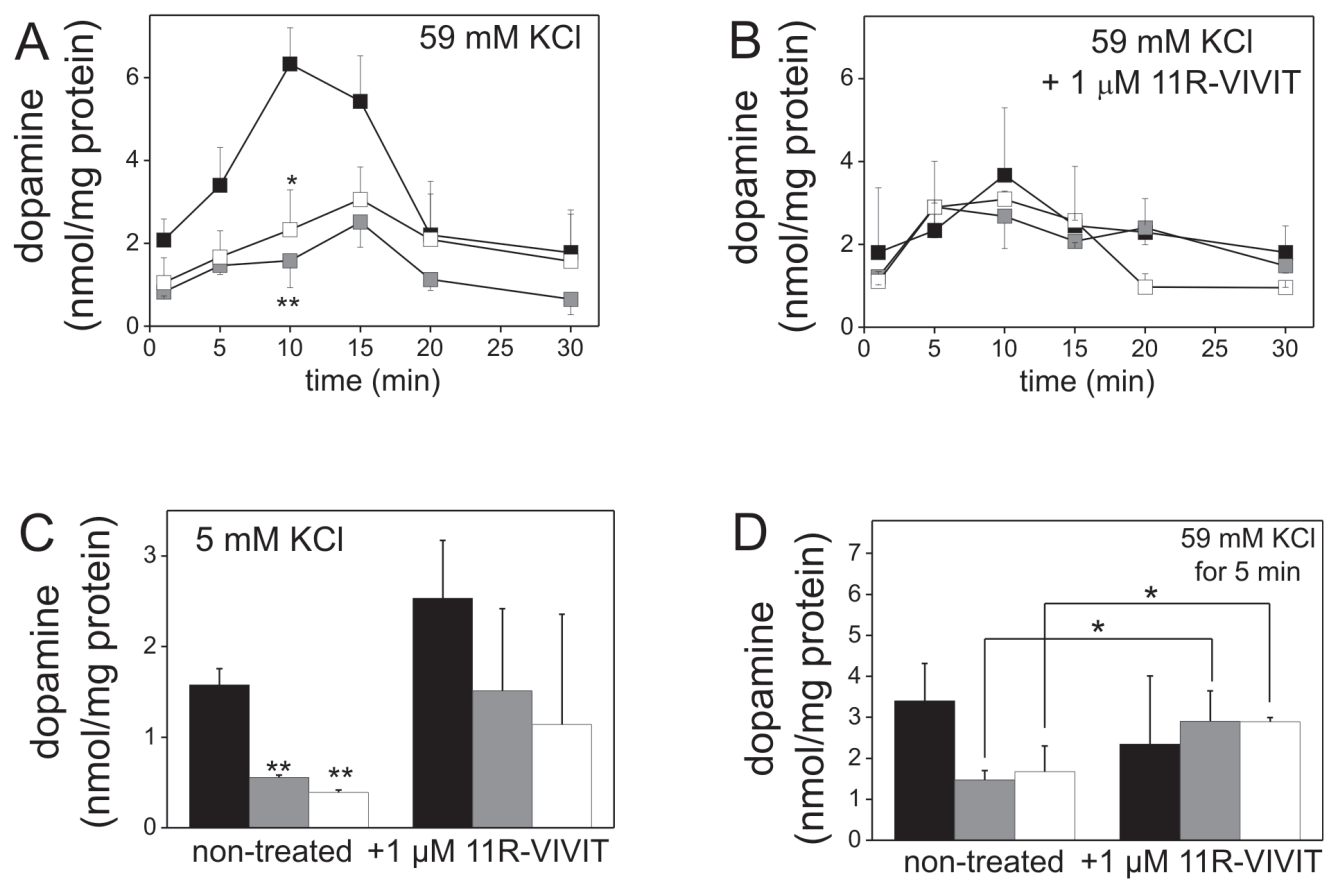
Effect of NFAT inhibition on dopamine secretion by PMCA2- or PMCA3-deficient PC12 cells. The amount of secreted dopamine was measured by RP-HPLC in samples obtained after 1, 5, 10, 15, 20, 30 min of stimulation with 59 mM KCl for non-treated cells (n = 6) (**A**) and for cells treated with NFAT inhibitor, 1 µM 11R-VIVIT (n = 3) (**B**). The amount of released dopamine at resting state (5 mM KCl) was compared between cells non-treated and treated with 1 µM 11R-VIVIT for 48 h (n>3) (**C**). Dopamine secreted after 5 min of stimulation was compared between cells non-treated and treated with 1 µM 11R-VIVIT for 48 h (n>3) using two-way ANOVA test (**D**). Bars represent mean values ± SEM. Student's t-test was used for comparison of control cells with PMCA2- or with PMCA3-reduced cells (A–C). *P≤0.05, **P≤0.01. Bars and symbols: filled – control cells (C), gray – PMCA2-deficient cells (_2), open – PMCA3-deficient cells (_3).

Abnormality of calcium homeostasis could affect the secretory activity of PC12 cells. To test this assumption we analyzed dynamics of dopamine secretion in PC12 cell stimulated with KCl. The obtained results revealed that dopamine is released with a significant delay by cells with altered pattern of PMCA isoforms in comparison to control. More precisely, the peak of dopamine secretion in control cells was observed after 10 min of stimulation, whereas in PMCA2- and PMCA3-deficient cells after 15 min (Fig. 4A). Moreover, inhibition of NFAT with 11R-VIVIT significantly reduced dopamine secretion in control cells (Fig. 4B). Conversely, in PMCA2- or PMCA3-deficient cells inhibition of NFAT resulted in stimulation of dopamine secretion already after 5 min of stimulation (Fig. 4B and Fig. 4D). It should be emphasized that under resting conditions treatment with 11R-VIVIT led to stimulation of dopamine secretion in all tested cell lines (control, PMCA2-, PMCA3-deficient cells) (Fig. 4C and 4D).

These results suggested that over-activation of NFAT in cells with reduced PMCA2 or PMCA3 content, both under resting and stimulating conditions, might modulate expression pattern of NFAT-dependent genes including those encoding proteins involved in secretory machinery. This, in turn, could lead to a delay and reduction of dopamine secretion. These data indicate indispensability of PMCA2 and PMCA3 for proper Ca^2+^/calcineurin/NFAT signaling.

### Impaired distribution of dopamine-containing vesicles in PMCA2- or PMCA3-deficient PC12 cells

**Figure 5:**
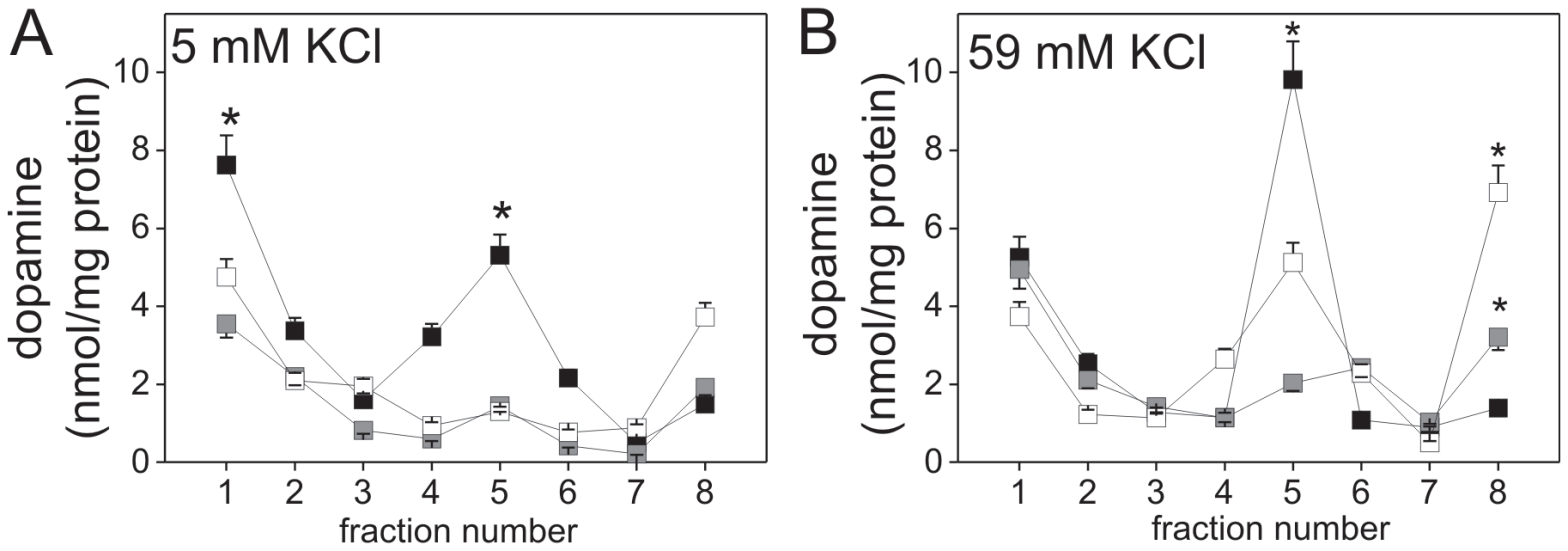
Dopamine distribution in vesicular fractions from PMCA2- or PMCA3-deficient PC12 cells. Dopamine content was determined by RP-HPLC in fractions obtained after subcellular fractionation from cells in resting conditions (**A**) or upon stimulation for 10 min with 59 mM KCl (**B**). Bars represent mean values±SEM. Student's t-test was used for comparison of the intracellular dopamine content in control cells with PMCA2- or PMCA3-reduced cells (n = 3). *P≤0.05, **P≤0.01. Bars and symbols: filled – control cells (C), gray – PMCA2-deficient cells (_2), open – PMCA3-deficient cells (_3).

To asses the mechanism responsible for reduced dopamine secretion in PMCA2- or PMCA3-deficient cells, subcellular distribution of dopamine in various types of secretory vesicles was tested. Fractionation in sucrose linear gradient allowed us to obtain several fractions that contained plasma membrane-docked vesicles (fractions 1–3), with α1-Na^+^/K^+^-ATPase as a protein marker, matured vesicles in the trans Golgi network (TGN) (fractions 4–5) with 58K and GM130 protein markers, and immature vesicles (fractions 6–8) with dopamine-β-hydroxylase protein marker. GM130 is a *cis*-Golgi matrix protein, present to some extent also in medial- and *trans*-Golgi [30]. Morphologically and by density the immature secretory vesicles could resemble dilated *trans*-Golgi cisternae that have pinched off the Golgi stack [31], thus it is possible that GM130-containg membranes, cisternae, or vesicles from the Golgi network may have been found in fractions 6–8, together with some immature vesicles. The low level of synaptophysin, a protein marker of small synaptic vesicles (SSV), revealed that the isolated fractions contained only very little amount of SSV (Fig. S2). RP-HPLC analysis of dopamine content showed that under resting conditions it was significantly lower in the plasma membrane-docked vesicles (fractions 1–3) and in matured vesicles in TGN (fractions 4–5) in PMCA2- or PMCA3-deficient cells than in control ones. This suggests a block of membrane fusion and a slower rate of biogenesis and repackaging of vesicles in TGN in PMCA2- or PMCA3-deficient cells (Fig. 5A). Upon stimulation, the secretion of dopamine stored in the plasma membrane-docked vesicles was significantly reduced in PMCA2- or PMC3-deficient cells, which were arrested at the stage of vesicle docking at the plasma membrane. This correlates with debilitation of vesicles docking and/or membrane fusion steps. Furthermore, dopamine amount in the TGN compartment was significantly elevated in stimulated control cells. This suggests an enhancement of sorting and packaging of the cargo into vesicles, but not in PMCA2- or PMCA3-deficient cells. Finally, the amount of dopamine in immature vesicles was significantly higher in stimulated PMCA2- and PMCA3-deficient cells than in the same cells under resting conditions. Moreover, it was also higher than in control cells (Fig. 5B). These observations suggest an enhancement in dopamine synthesis and packaging into immature vesicles in spite of limited secretion.

### Expression of genes encoding proteins involved in secretory pathway depends on NFAT transcriptional activity in PMCA2- or PMCA3-deficient PC12 cells

**Figure 6:**
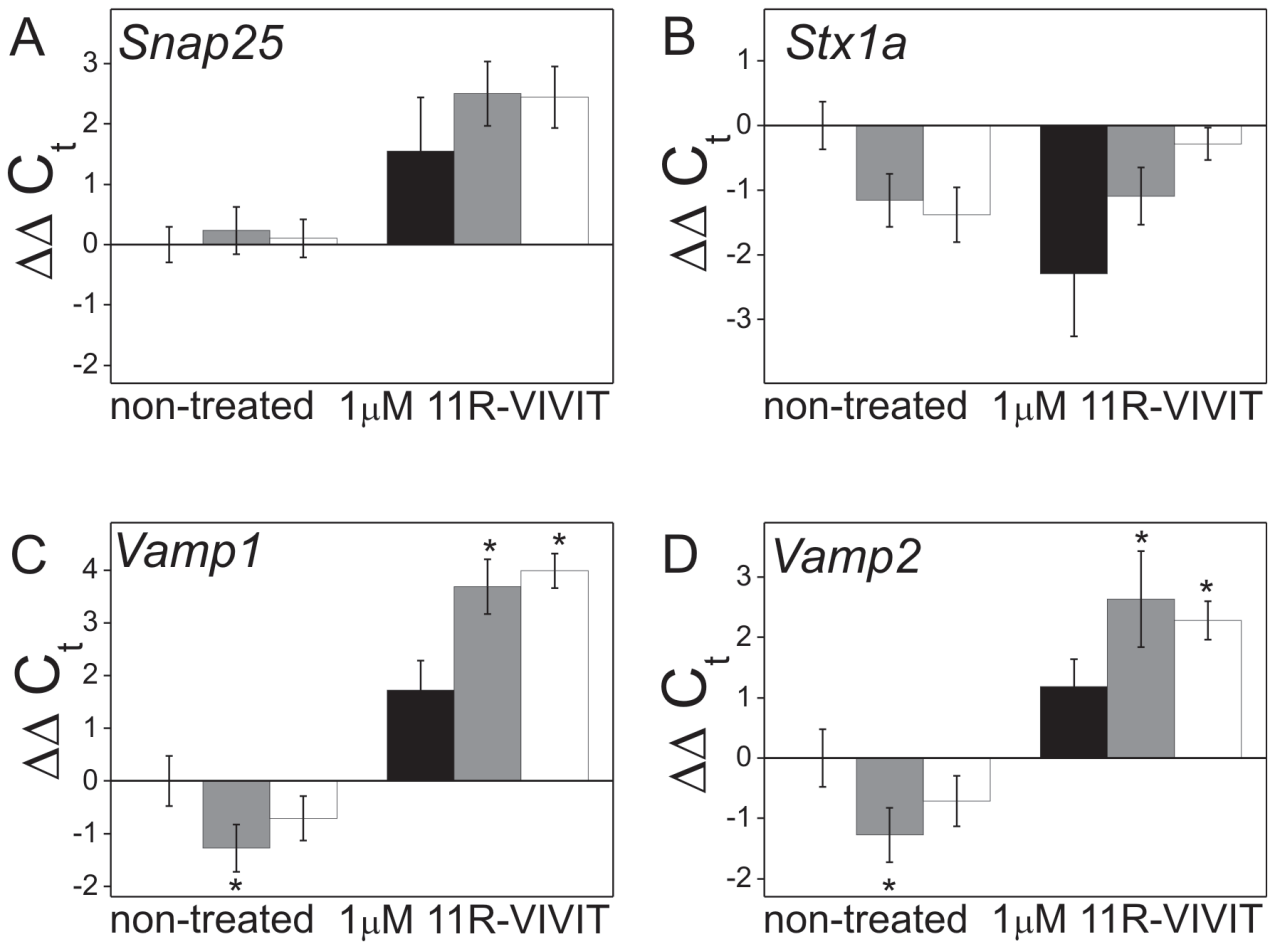
Effects of NFAT inhibition on expression of selected genes encoding proteins involved in secretion of dopamine in PMCA2- or PMCA3-deficient PC12 cells. RNA was isolated from non-treated cells or cells treated with 1 µM 11R-VIVIT for 48 h. Expression of genes encoding components of SNARE complex was examined by qPCR: *Snap25* (**A**), *Stx1a* (**B**), *Vamp1* (**C**), *Vamp2* (**D**). Bars represent mean values of ΔΔC_t_±SEM. Wilcoxon test for ΔC_t_ from qPCR data was used for comparison of control cells (ΔC_t_ standardized to y = 1) with PMCA2- or PMCA3-deficient cells (n = 3). *P≤0.05, **P≤0.01. Bars: filled – control, gray – PMCA2-deficient, open – PMCA3-deficient.

To test the hypothesis that NFAT is responsible for disturbed dopamine secretion by influencing the expression pattern of the secretory pathway genes, the effect of NFAT inhibition by 11R-VIVIT was investigated by qPCR. It was found that the content of SNAP25 (*Snap25*), an element of the SNARE membrane fusion complex, was similar in PMCA2- or PMCA3-deficient cells, non-treated with VIVIT (Fig. 6A, left). In contrast, the expression of other elements of the SNARE complex, i.e. syntaxin 1a (*Stx1a*) (Fig. 6B, left), VAMP1 (*Vamp1*) (Fig. 6C, left), and VAMP2 (*Vamp2*) (Fig. 6D, left) was lower in PMCA2 or PMCA3-deficient cells. A significant increase in the amount of the transcripts of SNAP25 (*Snap25*) (Fig. 6A, right), VAMP1 (*Vamp1*) (Fig. 6C, right) and VAMP2 (*Vamp2*) (Fig. 6D, right) was observed upon NFAT inhibition. The only exception was syntaxin 1a (*Stx1a*) (Fig. 6B, right), whose level was decreased.

**Figure 7:**
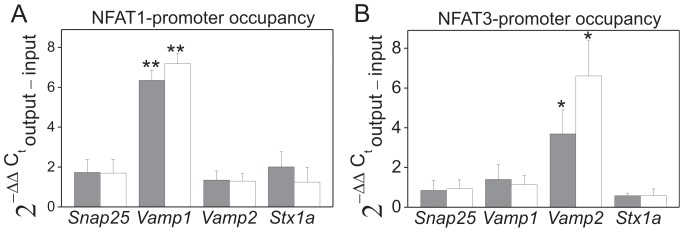
NFAT1 and NFAT3 binding to the promoters of genes encoding proteins involved in dopamine secretion in PMCA2- or PMCA3-deficient PC12 cells. The binding of NFATs to the promoter region was analyzed by chromatin immunoprecipitation-qPCR as described in methods. The following genes were analyzed: *Snap25*, *Vamp1*, *Vamp2*, *Stx1a*. Data are shown as the fold change (2^−ΔΔCt^ ±SEM) of the promoter occupancy by NFAT1 (**A**) and by NFAT3 (**B**). Wilcoxon test for ΔC_t_ from qPCR data was performed for comparison of the fold of change in NFAT promoter occupancy in control cells (C, standardized to y = 1) with PMCA2- (_2) or PMCA3-deficient cells (_3) (n = 4). *P≤0.05, **P≤0.01. Bars: gray – PMCA2-deficient cells, open – PMCA3-deficient.

Chromatin immunoprecipitation qPCR method demonstrated that in PMCA2- and PMCA3-deficient cells NFAT1 binds to the promoter region of *Vamp1* (Fig. 7A), while NFAT3 to the promoter region of *Vamp2* (Fig. 7B). The results were confirmed by subjecting the qPCR samples from ChIPs to electrophoresis on an agarose gel (Fig. S3).

### Impaired SNARE complex formation in PMCA2- or PMCA3-deficient cells

**Figure 8:**
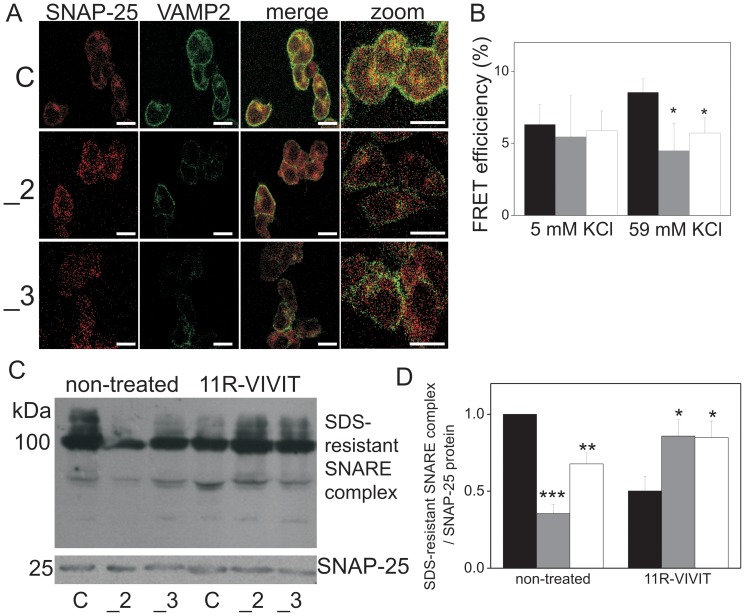
Formation of the SNARE complex in PC12 cells with reduced content of PMCA2 or PMCA3. Cellular distribution of SNAP-25 (Alexa Fluor 546 - red) and VAMP2 (Alexa Fluor 488 - green) (images represent cells stimulated with 59 mM KCl) (bar = 10 µm) was determined immunocytochemically (**A**). Microscopic analysis of SNAP-25 and VAMP2 interaction was performed by FRET efficiency measurements (n>5 images from each cell line) (**B**). The formation of the SDS-resistant SNARE complex was analyzed by immunoblotting. The 100 kDa SNAP-25 band represents the SDS-resistant SNARE form and the 25 kDa SNAP-25 band represents the soluble form (**C**). The intensity of bands was quantified densitometrically, standardized to soluble SNAP-25 and normalized to control cells, expressed as y = 1 (**D**). Bars represent mean values ± SEM. Student's t-test was used for comparison control cells with PMCA2- or PMCA3-reduced cells. *P≤0.05, **P≤0.01. Bars and symbols: filled – control cells (C), gray – PMCA2-deficient cells (_2), open – PMCA3-deficient cells (_3).

Finally, to characterize the physiological implications of the aforementioned effects of PMCA2 or PMCA3 deficiency on the formation of the SNARE complex the latter was analyzed by microscopic fluorescence energy transfer (FRET) method. These experiments revealed a reduced energy transfer between fluorochromes bound to SNAP-25 and VAMP2 in PMCA2- or PMCA3-deficient cells (a weaker fluorescence signal from VAMP2-Alexa Fluor 488 upon bleaching of SNAP-25-Alexa Fluor 546). This suggests lower degree of co-localization of SNAP-25 and VAMP2 (Fig. 8A). This finding was confirmed by quantification of FRET efficiency between SNAP-25 and VAMP2, showing a significantly lower SNAP-25-VAMP2 binding in PMCA2- or PMCA3-deficient cells upon stimulation with KCl (Fig. 8B). The formation of a SDS-resistant SNARE complex was assessed by detection of a 100 kDa protein band corresponding to the SNARE complex and a 25 kDa protein band, corresponding to soluble SNAP-25 [32]. This analysis showed that the amount of the 100 kDa SDS-resistant SNARE complex was significantly decreased in PMCA2- and PMCA3-deficient cells in comparison to control, while the content of soluble 25 kDa SNAP-25 protein was stable in all cell lines tested. Finally, the treatment with 11R-VIVIT, blocking NFAT, restored the formation of the 100 kDa SNARE complex in PMCA2- and PMCA3-deficient cells (Fig. 8C), as confirmed by densitometric analysis (Fig. 8D). Moreover, altered subcellular distribution of SNAP-25 and VAMP2 reinforced the notion of an impaired formation of the SNARE complex in PMCA2- or PMCA3-deficient cells. Subcellular distribution of VAMP2 suggested a significant drop in the amount of VAMP2 in the TGN compartment in PMCA2- and PMCA3-deficient PC12 cells both in unstimulated and stimulated cells. This was followed by a block of VAMP2 translocation towards plasma membrane (fractions 1–3) upon stimulation. These data were quantified densitometrically confirming affected cellular distribution of SNARE components in PMCA2- or PMCA3-deficient cells (Fig. S4).

## Discussion

Abnormally increased [Ca^2+^]_c_ in the dopaminergic type of pheochromocytoma tumor results in excessive secretion of dopamine [1]. PMCA2 and PMCA3 isoforms, highly expressed in pheochromocytoma cells, assure fast and efficient calcium extrusion during tumor development [5], [7], preventing prolonged increase in dopamine secretion. In view of this fact one could expect that deficiency in these isoforms should increase dopamine secretion. Surprisingly, in this study we showed that downregulation of PMCA2 or PMCA3 isoforms in PC12 cells resulted in a significant decrease in dopamine secretion despite increased [Ca^2+^]_c_ under resting conditions and intensified calcium response upon stimulation. This observation sheds new light on the roles of particular PMCA isoforms in the regulation of secretory activity of PC12 cells. The importance of various PMCAs in different types of secretory response has been already suggested in the case of lymphocytes [33], osteoclasts [34], mammary gland [35], during insulin release [36], and islets of Langerhans and pancreatic β-cell lines [37].

We have observed that changes in calcium homeostasis were accompanied by over-activation of NFAT as demonstrated by altered NFAT cellular localization, increased level of dephosphorylated active form and finally by an increased activity, verified by luciferase reporter assays. Data shown here indicate that NCX does not compensate for the effects of reduced PMCA2 and PMCA3 activity in PC12 cells under resting conditions. Such compensation was suggested as a result**s** of NCX and PMCA tight cooperation leading to the maintenance of low and balanced [Ca^2+^]_c_ [38]. Neither the use of NCX inhibitor (KB-R7943), nor the absence of sodium ions, influenced resting [Ca^2+^]_c_. Thus, we conclude that the only factor responsible for changes in calcium handling and calcium dependent cell signaling at the resting conditions was the reduction in PMCA2 and PMCA3 content.

Nevertheless, regarding an increase in the expression of NCX and the alleged NCX contribution during cell stimulation with 59 mM KCl, it should be taken into account that NCX might be important for signaling processes and gene expression regulation upon plasma membrane depolarization. Although we did not test how KB-R7943 treatment may influence NFAT activation upon cell stimulation with 59 mM KCl, we discuss this issue based on the literature data. Firstly, a store-operated Ca^2+^ entry, influencing directly the NFAT/calcineurin pathway was not inhibited with KB-R7943 in cardiomyocytes exhibiting an increased calcineurin-NFAT activation [39]. In addition, an inhibitor of NCX, KB-R7943, did not reduce the NFAT-mediated Ca^2+^ entry in myocytes [40]. Conversely, other data suggested that NCX may contribute to the NFAT-dependent excitation–transcription coupling in the heart [41]. Additionally, NCX activity was found to be regulated by calcineurin [42]–[44], and NCX expression pattern was found to be under influence of calcineurin [45], [46]. This data suggest that NCX contribution to NFAT signaling upon cell stimulation is possible but complex and might vary depending on cell types and the processes involved. This points to the role of particular PMCA isoforms in calcineurin/NFAT signaling in neuroendocrine cells. However, it would be interesting to test in the future activation of NFAT upon KB-R7943 treatment and upon plasma membrane depolarization in PC12 cells.

Furthermore, we tested the potential contribution of VDCC to calcium signaling and, putatively, to modulation of gene expression via NFAT-dependent mechanisms. According to the mechanism of VDCC action, its role in the rise of [Ca^2+^]_c_ under resting conditions is very unlikely. Yet, based on the observed increase in the plasma membrane potential under resting conditions (as measured by patch clamp, results not shown) it becomes probable that some VDCCs could be already open at rest and thus contribute to a leakage of calcium ions into the cell. Nonetheless, inhibition of VDCC did not alter [Ca^2+^]_c_ at resting state, excluding its involvement in the observed increase in [Ca^2+^]_c_ during resting conditions. The increased content of L-type VDCC, and also of T-type VDCC, in cells with altered PMCA composition is difficult to be interpreted. This may be linked with possible VDCC contribution to calcineurin/NFAT signaling during secretory response triggered by plasma membrane depolarization. Nevertheless, our data indicate that enhanced calcium signaling was not followed by appropriate secretion of dopamine; on the contrary, dopamine secretion by cells with downregulated PMCA2 or PMCA3 deteriorated significantly. In this study we did not test the effect of nifedipine on NFAT activation in stimulated cells. However, on the basis of literature it could be assumed that pharmacological inhibition of VDCC decreases NFAT activity during cell stimulation. For instance, it has been found that nifedipine prevented activation of NFAT by phenylephrine in cardiac hypertrophic myocytes [47]. Moreover, it has been demonstrated that L-type VDCC blockers and cyclosporine A (calcineurin inhibitor) act additively to suppress the Ca^2+^-calcineurin-NFAT signaling pathway [48]. Nifedipine has also blocked Ca^2+^-induced NFAT nuclear translocation suggesting that Ca^2+^ influx through L-VDCC might be a primary source of calcium ions for activation of calcineurin-NFAT signaling in ventricular myocytes [49]. Furthermore, activity-dependent translocation of NFAT1 has been found to be strictly dependent on the activation of N-type calcium channels since this effect was blocked by ω-conotoxin GVIA, a specific N-type channel inhibitor [50]. It was also shown that depolarization of neurons induced activation of NFAT3 [51], and the effect was linked directly with L-type VDCC, but not with N- and P/Q-types of VDCC [52]. In addition, anchoring of calcineurin to L-type VDCC is required for activation of the NFAT3-dependent gene regulation pathway [53]. These examples demonstrate that VDCCs can be involved in gene regulation by calcium-sensing elements, including the calcineurin/NFAT pathway [54]. To summarize, the activity of NFAT might be linked with a VDCC-mediated Ca^2+^ influx, as has been proven especially in muscle cells. Moreover, an increased expression and protein content of VDCC (L and T type) in the cells with over-activated NFAT might be in a close relationship with a secretory response observed after increase in KCl concentration in the medium.

We showed that despite increased [Ca^2+^]_c_ dopamine secretion was substantially reduced in PMCA2- or PMCA3-deficient PC12 cells. This is particularly surprising because the enhancement of [Ca^2+^]_c_ was accompanied by activation of NFAT1 and NFAT3, already under resting conditions. It may be hypothesized that PMCA2 and PMCA3 have a special role in modulation of NFAT-dependent calcium signaling which is necessary for proper gene regulation during exocytosis. Accordingly, the main hypothesis tested in this study was NFAT contribution to the regulation of secretory response in neuroendocrine cells. In fact, little is known about the role of NFAT in catecholamine secretion from neuroendocrine cells. So far it has been demonstrated that Ca^2+^/calcineurin-dependent activation of NFAT is important in many processes including: neuronal cell differentiation [55], axonal growth, and neuronal development [56]. Moreover, NFAT has been shown to participate in the regulation of expression of genes encoding proteins involved in secretory processes such as: depolarization induced growth hormone release from anterior pituary gland [57], β-cell endocrine function [58], catecholamine synthesis by chromaffin cells induced by the corticotrophin-releasing factor (CRF) pathway [59]. The mechanism of NFAT-dependent gene expression regulation has been described in the case of T-cells activation and, especially, lytic granule exocytosis in NK-92 natural killers [60]. A similar regulatory mechanism has been proposed in several studies pointing to indirect involvement of PMCA isoforms in regulation of the calcineurin/NFAT pathway [61], [62]. Overall, these data support our findings that NFAT may be indeed involved in rearrangements of secretory pathway genes expression pattern in pheochromocytoma cells, especially, upon altered calcium signaling due to the lack of indispensable PMCA2 and PMCA3 isoforms.

At this point, it should be added that our results and the conclusions drawn from them are based on the experiments with a the use of a common NFAT inhibitor, 11R-VIVIT. This molecule is a modified regulatory peptide (RRRRRRRRRRR-GGG-MAGPHPVIVITGPHEE), naturally located at the N′-terminus of NFAT. 11R-VIVIT prevents a conformational change and inhibits further NFAT binding to the target DNA sequences [63]. 11R-VIVIT masks the calcineurin-docking site in NFAT and further blocks the translocation of NFAT from cytosol to the nucleus [64]. 11R-VIVIT induced inhibition of NFAT action was observed in neurons and glial cells in Alzheimer's disease [65]. Moreover, according to the literature, 11R-VIVIT was found to efficiently block transcriptional activity of NFAT, manifested by an arrested NFAT translocation [66], decreased NFAT expression in osteoclast differentiation [67], and by alterations of the expression pattern of genes being under its control [68], [69].

In this report we focused on the role of two NFAT isoforms in dopamine secretion. We analyzed a ubiquitous isoform, NFAT1, mainly involved in regulation of interleukin and chemokine secretion by lymphocytes [70] and in regulation of the contraction process in cardiomyocytes [71], and a neural NFAT3 isoform, present at the highest level in cortex, hippocampus, and the spinal cord and involved in regulation of axon growth and neuronal death [19]–[21]. We propose direct involvement of NFAT isoforms in regulation of the exocytosis pathway genes during dopamine secretion by pheochromocytoma cells. We conclude that over-activation of NFAT might be linked to the reduction of PMCA2 and PMCA3 level, and thus to altered [Ca^2+^]_c_. Furthermore, we propose that activation of NFAT1 and NFAT3 might result in the repression of *Vamp1* and *Vamp2*, respectively. Since these genes encode synaptobrevin 1 and synaptobrevin 2, that are crucial for proper assembly of the SNARE complex [72], it demonstrates that NFAT signaling regulates exocytosis by limiting the membrane fusion step. The observed perturbation in SNARE complex formation was reversed by treatment with NFAT inhibitor. Moreover, we found NFAT binding motifs in the sequences of *Vamp1* and *Vamp2* genes. The same motifs were already reported as repressor elements downstream the FIRE (Fast Intronic Element Controls) sequences, involved in inhibition of genes during muscle development and muscle contraction [73]. The inhibitory influence of NFATs on *Vamp* expression proposed in this work is supported by several research reports providing evidence on the repressive role of NFATs. NFATs played an inhibitory role in gene induction during neuronal development [56], neuronal survival [19] and chondrogenesis [74]. NFAT does not act alone. It cooperates with histone deacetylases (HDAC), repressing gene expression during osteoblast differentiation [75]. This gives a direction for future studies.

Finally, putative functional differences between NFAT1 and NFAT3 should also be discussed. NFAT inhibition with 11R-VIVIT led to a block in KCl-mediated dopamine secretion in control cells, suggesting that stable amount and activity of NFAT is indispensable for the activation of dopamine secretion. Thus, destabilization of an NFAT-dependent regulatory mechanism may be followed by other consequences related to transcriptomic instability. Consequently, we suggest that increased levels of NFAT1 and NFAT3 in PMCA2- or PMCA3-deficient cells, could have a negative impact on expression of genes crucial for dopamine secretion. Based on different content of NFAT1 and NFAT3 and on their different target gene (VAMP1 and VAMP2, respectively), it could be concluded that these two members of the NFAT family play distinct roles in dopamine secretion in PC12 cells. Regarding the fact that NFAT3 was found to influence numerous neuro-specific processes, including neuronal axons outgrowth, neural development, neural apoptosis [19]–[21], it is very likely that NFAT3, but not NFAT1, would be specifically involved in regulation of VAMP2, which is a common protein engaged in neurotransmitter release and involved in neurite elongation [76], endo-lysosomal degradation and prevention of neurodegeneration [77]. These facts suggest that various mechanisms regulating the intracellular trafficking process may have similar requirements - one dependent on the ubiquitous NFAT1, and the second dependent on the neuron-specific NFAT3.

## Conclusions

Activation of NFAT has been suggested to be necessary for catecholamine synthesis by chromaffin cells with the involvement of the CRF pathway [59]. On the other hand, important data suggesting that PMCA expression could be induced by NFAT1 in osteoclasts during the growth of bone mass have been provided recently [34]. Furthermore, Kim *et al.* [34] have shown that PMCA-mediated increase in Ca^2+^ efflux prevented NFAT1 activation, forming a negative regulatory mechanism for the regulation of PMCA expression pattern. In this study, we suggest a novel regulatory mechanism linking NFAT with PMCA-dependent calcium signaling pathway and dopamine secretion. We have shown that the presence of PMCA2 and PMCA3 isoforms seems to be crucial for calcineurin/NFAT signaling which regulates dopamine secretion by inhibiting expression of *Vamp* genes encoding proteins involved in exocytosis of neurotransmitters. Finally, by demonstrating that abnormal NFAT activation, due to altered composition of PMCAs, could influence the secretion of one of the catecholamines, dopamine, we postulate a new role of NFAT in a variety neurological pathologies.

## Supporting Information

Figure S1 **Expression pattern of NCX and VDCC.** The expression of *Slc8a1* (NCX1), *Slc8a2* (NCX2) and *Slc8a3* (NCX3) in PC12 cells was determined by qPCR (**A**). Similarly, the expression of different types of VDCC was also tested by qPCR: *Cacna1c* (L type α1c), *Cacna1d* (L type α2d), *Cacna1b* (N type), *Cacna1a* (P/Q type) and *Cacna1h* (T type) in PC12 cells (**B**). The protein content of NCXs (NCX1 and NCX3) (according to accessible antibodies) was verified by immunoblotting (**C**). The protein content of VDCCs (L type α1c, L type α2d, N type, P/Q type and T type) was verified by immunoblotting (**D**). The immunoblots were standardized to GAPDH and normalized to control cells, expressed as y = 1, both for total NCX content (**E**) and all VDCC types (**F**). Bars represent mean values ± SEM. Student's t-test was used in the densitometry for comparison of control cells with PMCA2- or PMCA3-reduced cells (n = 4). Wilcoxon test was used for ΔC_t_ from qPCR data (n = 3) for comparison of control cells (ΔC_t_ expressed as y = 0) with PMCA2- or PMCA3-reduced cells (n = 3).*P≤0.05, **P≤0.01. Bars and symbols: filled – control cells (C), gray – PMCA2-deficient cells (_2), open – PMCA3-deficient cells (_3).
(TIF)

Figure S2 **Subcellular distribution of protein markers in fractionated PMCA2- or PMCA3-deficient PC12 cells.** The fractions obtained by sucrose gradient centrifugation were characterized by immunoblotting in terms of subcellular protein marker distribution; p38 (synaptophysin) (small synaptic vesicles) Na^+^/K^+^-ATPase (plasma membrane), 58K (Golgi apparatus), GM130 (cis-region of Golgi apparatus), Rab3A and dopamine β-hydroxylase (DBH) (immature secretory granules), both under resting (5 mM KCl) and depolarizing (59 mM KCl) condition (**A**). The linearity of sucrose gradient was verified under resting (5 mM KCl) and depolarizing (59 mM KCl) conditions (**B**). Signs and symbols: filled – control cells (C), gray – PMCA2-deficient cells (_2), open – PMCA3-deficient cells (_3).
(TIF)

Figure S3 **NFAT binding to the promoter region of selected genes encoding elements of SNARE complex (** ***Snap25, Vamp1, Vamp2, Stx1a*** **) in PMCA2- or PMCA3-deficient PC12 cells.** The ChIP-qPCR results were confirmed by subjecting the qPCR samples (as negative control) to electrophoresis in 3% agarose gel in TAE buffer (pH 8.0) for 1 h. Symbols: control cells (C), PMCA2-deficient cells (_2), PMCA3-deficient cells (_3).
(TIF)

Figure S4 **Changes of subcellular distribution of SNAP-25 and VAMP2 in PMCA2- or PMCA3-deficient PC12 cells under resting and stimulating conditions.** The subcellular re-location of SNAP-25 and VAMP2 was examined by immunoblotting of sucrose fractions isolated from cells maintained in resting conditions (5 mM KCl) or subjected to plasma membrane depolarization (59 mM KCl) (A). Densitometric analysis of VAMP2 and SNAP-25 distribution in the fractions was performed as follows: the immunoblotted bands were scanned, quantified and standardized according to the most intense band in the control cells, separately in resting and stimulating conditions (B). Signs and symbols: filled – control cells (C), gray – PMCA2-deficient cells (_2), open – PMCA3-deficient cells (_3).
(TIF)

Figure S5 **Apoptosis and cell cycle analysis of PC12 cells.** The cells were incubated in Locke's solution containing 5 mM KCl (resting conditions) for 30 min (A) containing 59 mM KCl (depolarizing conditions) for 30 min (B) or in the presence of 1 mM 11R-VIVIT for 48 h (C) were analyzed for apoptosis index and cell cycle using the Nicoletti's staining with propidium iodide by flow cytometry. Bars represent mean values ±SEM, n>3. Bars: black - control cells (C), gray - PMCA2-deficient cells (_2), white – PMCA3-deficient cells (_3); striped bars represent cell lines treated with 11R-VIVIT and no-striped bars represent cell lines incubated in the presence of 0.1% DMSO as control conditions.
(TIF)

Figure S6 **Alternative splicing of Atp2b2 (PMCA2) in PMCA2- or PMCA3-deficient PC12 cells.** Alternative splicing pattern at sites A and C of mRNA transcripts of Atp2b2 (PMCA2) was determined by RT-PCR according to Kamagate et al. 2000 [27]. Symbols: control cells (C), PMCA2-deficient cells (_2), PMCA3-deficient cells (_3).
(TIF)
