# Supplementary figures and images for: Calcineurin/NFAT Signaling Represses Genes Vamp1 and Vamp2 via PMCA-Dependent Mechanism during Dopamine Secretion by Pheochromocytoma Cells
Source: PLoS One. 2014 Mar 25;9(3):e92176. doi: 10.1371/journal.pone.0092176 (PMC3965406; doi:10.1371/journal.pone.0092176)

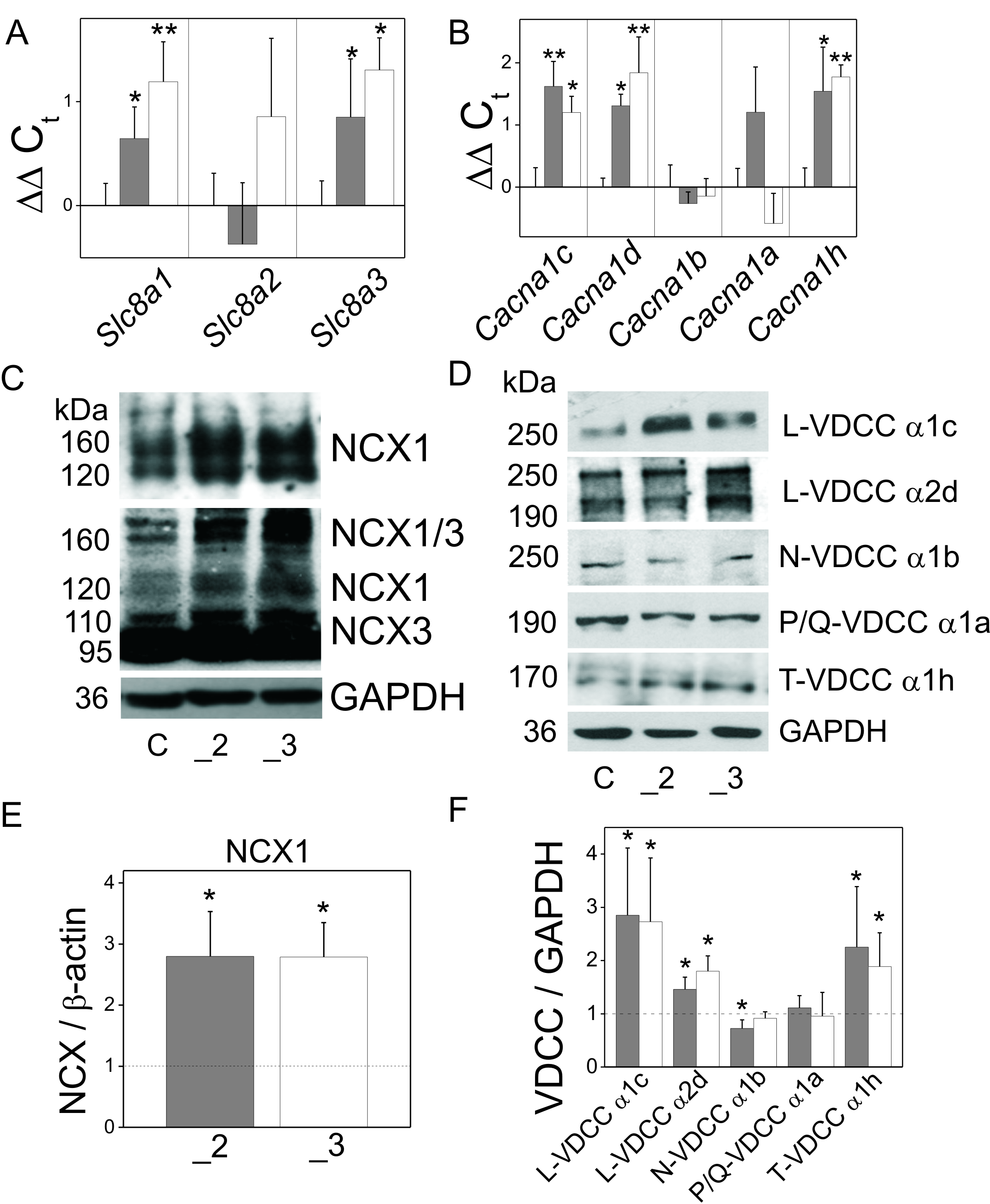

Supplement: Figure S1 — Expression pattern of NCX and VDCC. The expression of Slc8a1 (NCX1), Slc8a2 (NCX2) and Slc8a3 (NCX3) in PC12 cells was determined by qPCR (A). Similarly, the expression of different types of VDCC was also tested by qPCR: Cacna1c (L type α1c), Cacna1d (L type α2d), Cacna1b (N type), Cacna1a (P/Q type) and Cacna1h (T type) in PC12 cells (B). The protein content of NCXs (NCX1 and NCX3) (according to accessible antibodies) was verified by immunoblotting (C). The protein content of VDCCs (L type α1c, L type α2d, N type, P/Q type and T type) was verified by immunoblotting (D). The immunoblots were standardized to GAPDH and normalized to control cells, expressed as y = 1, both for total NCX content (E) and all VDCC types (F). Bars represent mean values ± SEM. Student's t-test was used in the densitometry for comparison of control cells with PMCA2- or PMCA3-reduced cells (n = 4). Wilcoxon test was used for ΔCt from qPCR data (n = 3) for comparison of control cells (ΔCt expressed as y = 0) with PMCA2- or PMCA3-reduced cells (n = 3).*P≤0.05, **P≤0.01. Bars and symbols: filled – control cells (C), gray – PMCA2-deficient cells (_2), open – PMCA3-deficient cells (_3). (TIF) [file pone.0092176.s001.tif]

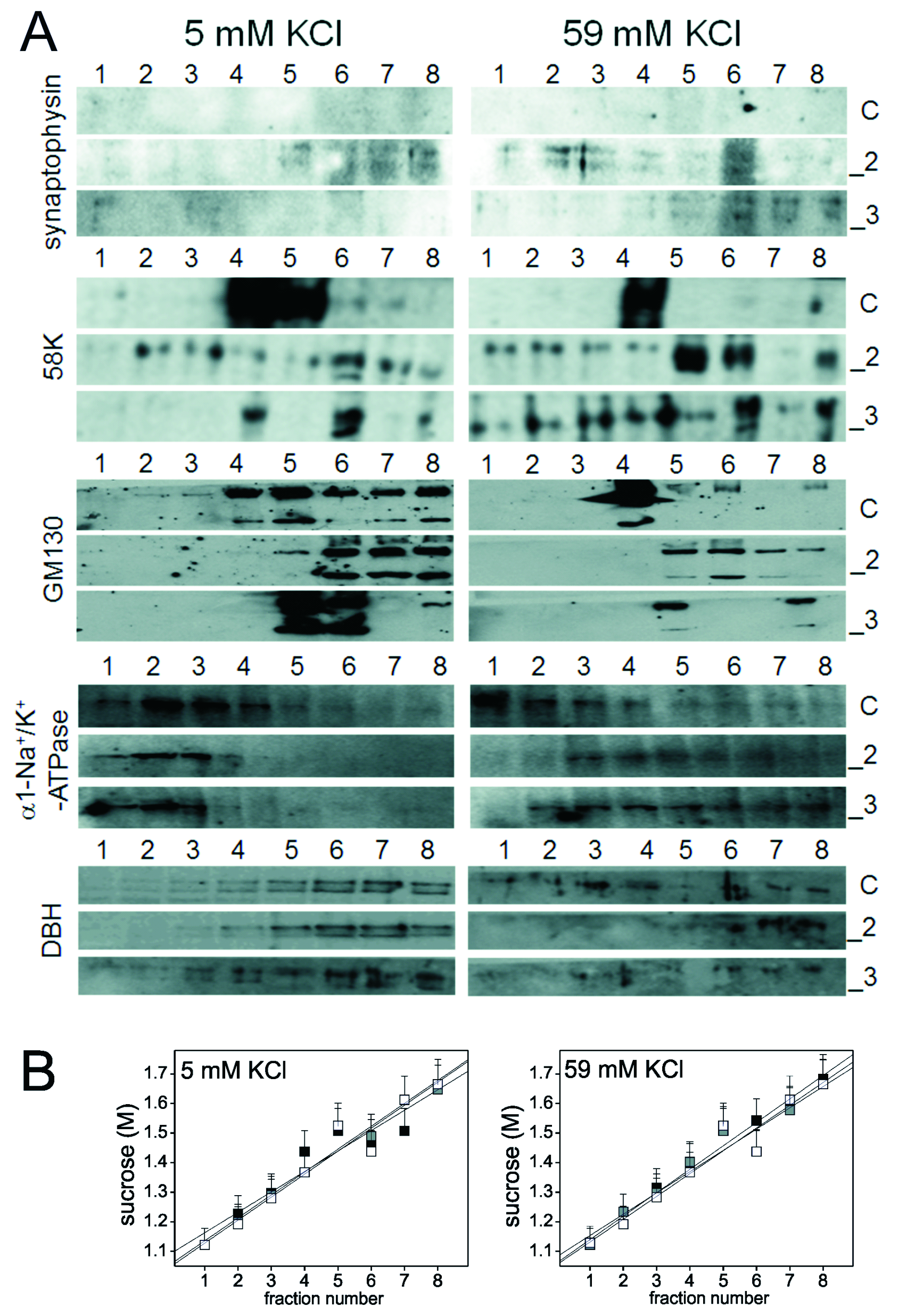

Supplement: Figure S2 — Subcellular distribution of protein markers in fractionated PMCA2- or PMCA3-deficient PC12 cells. The fractions obtained by sucrose gradient centrifugation were characterized by immunoblotting in terms of subcellular protein marker distribution; p38 (synaptophysin) (small synaptic vesicles) Na+/K+-ATPase (plasma membrane), 58K (Golgi apparatus), GM130 (cis-region of Golgi apparatus), Rab3A and dopamine β-hydroxylase (DBH) (immature secretory granules), both under resting (5 mM KCl) and depolarizing (59 mM KCl) condition (A). The linearity of sucrose gradient was verified under resting (5 mM KCl) and depolarizing (59 mM KCl) conditions (B). Signs and symbols: filled – control cells (C), gray – PMCA2-deficient cells (_2), open – PMCA3-deficient cells (_3). (TIF) [file pone.0092176.s002.tif]

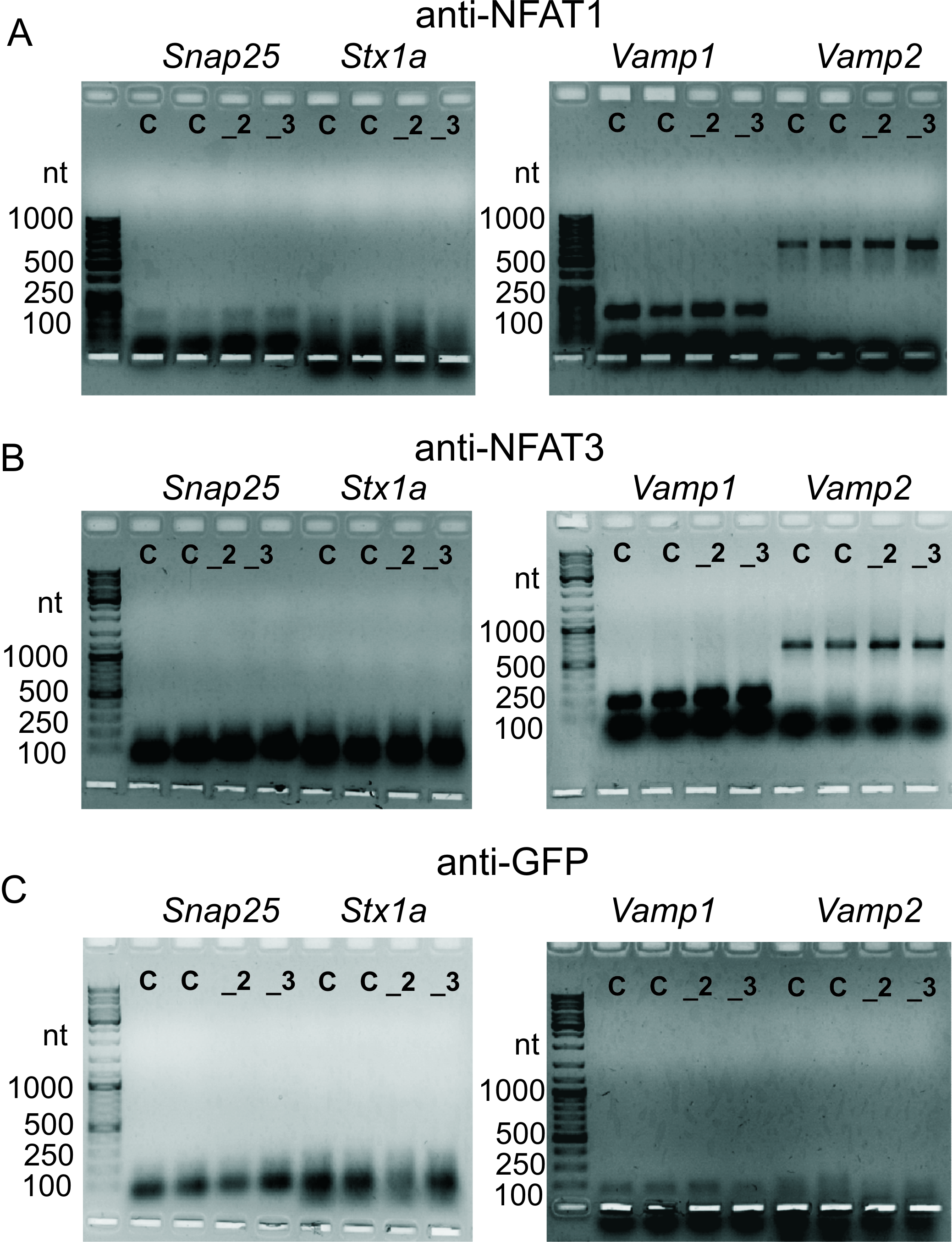

Supplement: Figure S3 — NFAT binding to the promoter region of selected genes encoding elements of SNARE complex ( Snap25, Vamp1, Vamp2, Stx1a ) in PMCA2- or PMCA3-deficient PC12 cells. The ChIP-qPCR results were confirmed by subjecting the qPCR samples (as negative control) to electrophoresis in 3% agarose gel in TAE buffer (pH 8.0) for 1 h. Symbols: control cells (C), PMCA2-deficient cells (_2), PMCA3-deficient cells (_3). (TIF) [file pone.0092176.s003.tif]

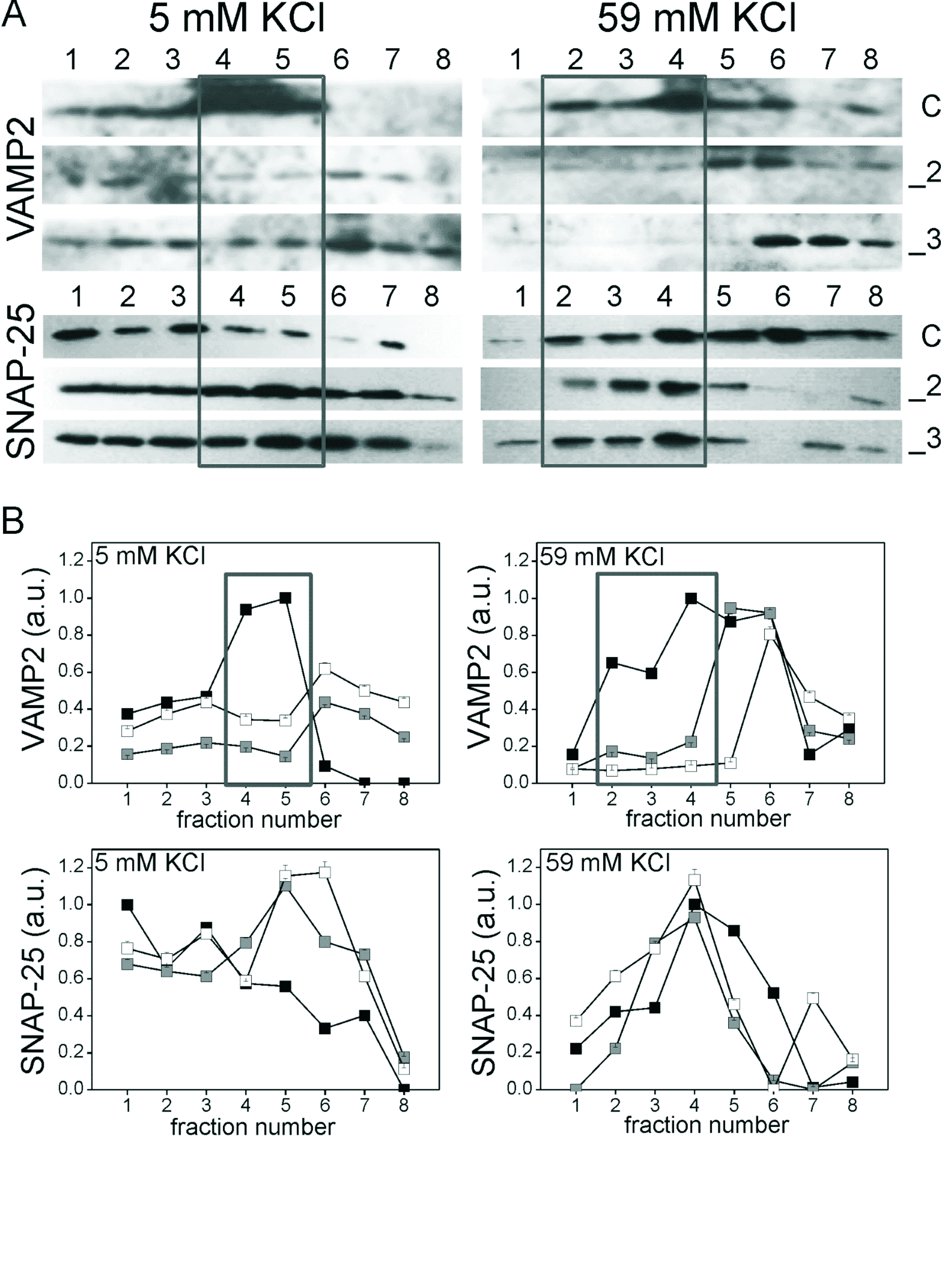

Supplement: Figure S4 — Changes of subcellular distribution of SNAP-25 and VAMP2 in PMCA2- or PMCA3-deficient PC12 cells under resting and stimulating conditions. The subcellular re-location of SNAP-25 and VAMP2 was examined by immunoblotting of sucrose fractions isolated from cells maintained in resting conditions (5 mM KCl) or subjected to plasma membrane depolarization (59 mM KCl) (A). Densitometric analysis of VAMP2 and SNAP-25 distribution in the fractions was performed as follows: the immunoblotted bands were scanned, quantified and standardized according to the most intense band in the control cells, separately in resting and stimulating conditions (B). Signs and symbols: filled – control cells (C), gray – PMCA2-deficient cells (_2), open – PMCA3-deficient cells (_3). (TIF) [file pone.0092176.s004.tif]

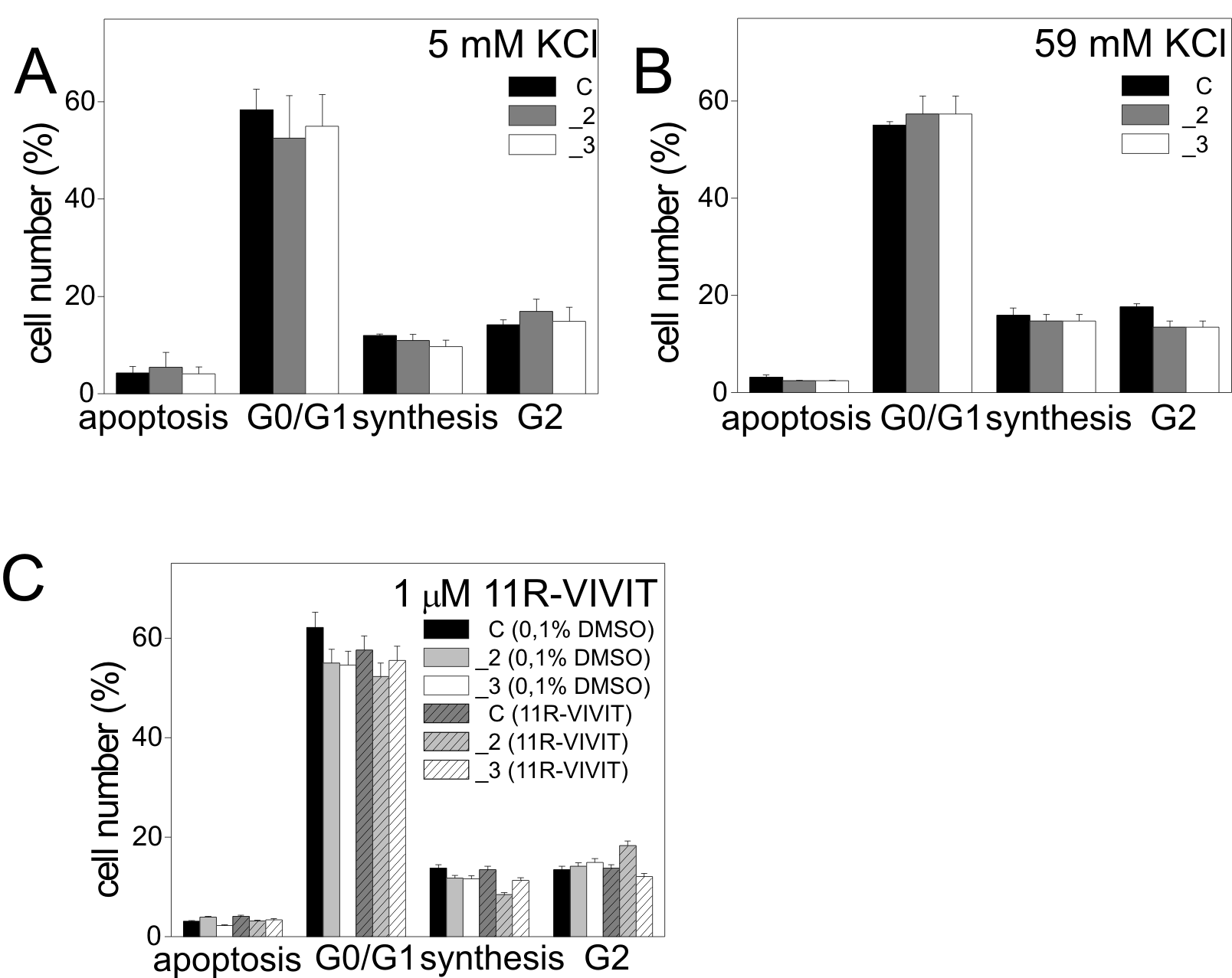

Supplement: Figure S5 — Apoptosis and cell cycle analysis of PC12 cells. The cells were incubated in Locke's solution containing 5 mM KCl (resting conditions) for 30 min (A) containing 59 mM KCl (depolarizing conditions) for 30 min (B) or in the presence of 1 mM 11R-VIVIT for 48 h (C) were analyzed for apoptosis index and cell cycle using the Nicoletti's staining with propidium iodide by flow cytometry. Bars represent mean values ±SEM, n>3. Bars: black - control cells (C), gray - PMCA2-deficient cells (_2), white – PMCA3-deficient cells (_3); striped bars represent cell lines treated with 11R-VIVIT and no-striped bars represent cell lines incubated in the presence of 0.1% DMSO as control conditions. (TIF) [file pone.0092176.s005.tif]

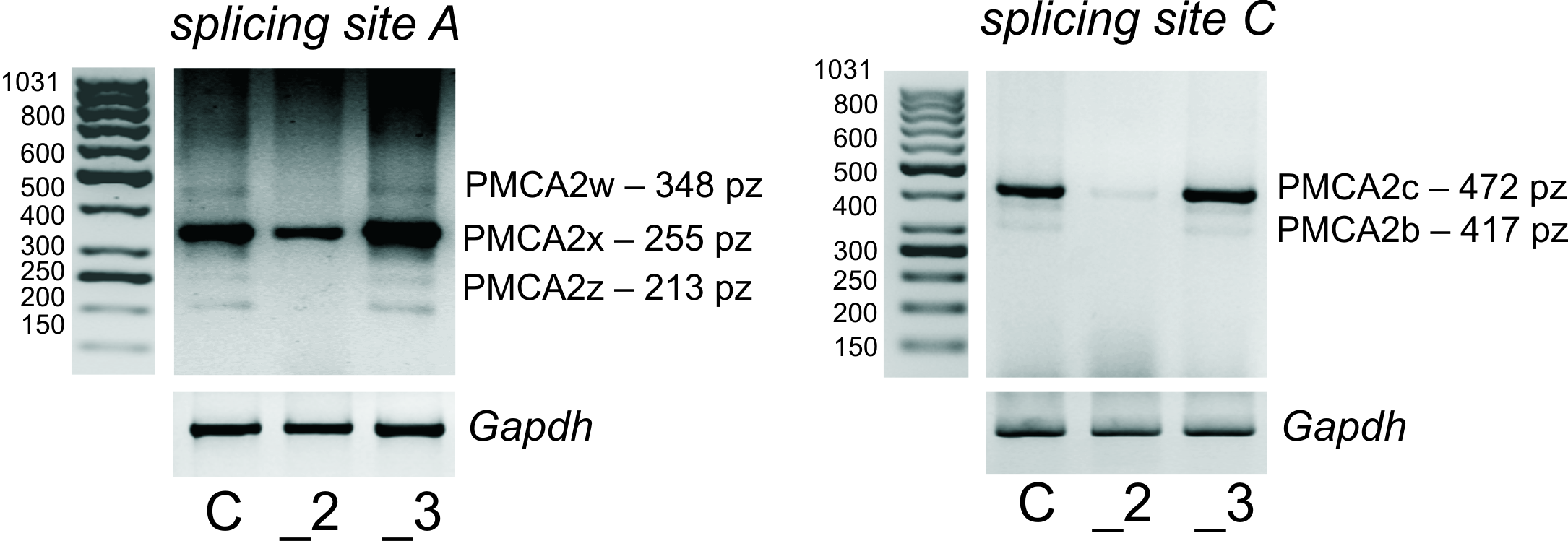

Supplement: Figure S6 — Alternative splicing of Atp2b2 (PMCA2) in PMCA2- or PMCA3-deficient PC12 cells. Alternative splicing pattern at sites A and C of mRNA transcripts of Atp2b2 (PMCA2) was determined by RT-PCR according to Kamagate et al. 2000 [27]. Symbols: control cells (C), PMCA2-deficient cells (_2), PMCA3-deficient cells (_3). (TIF) [file pone.0092176.s006.tif]
